# Radionuclide-Labeled Biomaterials: A Novel Strategy for Tumor-Targeted Therapy

**DOI:** 10.3390/biomimetics10060394

**Published:** 2025-06-11

**Authors:** Shu Zhang, Aiyue Zhang, Xunhao Qi, Zongtai Han, Luqi Song, Jiayu Zhou, Guanglin Wang, Ran Zhu, Jianguo Li

**Affiliations:** 1 CNNC Key Laboratory on Radio-Toxicology and Radiopharmaceutical Preclinical Evaluation, National Atomic Energy Agency Nuclear Technology (Nonclinical Evaluation of Radiopharmaceuticals) Research and Development Center, China Institute for Radiation Protection, Taiyuan 030006, China; zs19882024@163.com (S.Z.); hanzongtai96@163.com (Z.H.); zhoujiayu0720@163.com (J.Z.); 2 State Key Laboratory of Radiation Medicine and Protection, School of Radiation Medicine and Protection, Collaborative Innovation Center of Radiation Medicine of Jiangsu Higher Education Institutions, Soochow University, Suzhou 215123, China; aiyuez@163.com (A.Z.); xunhaoqi@163.com (X.Q.); 20244220079@stu.suda.edu.cn (L.S.); glwang@suda.edu.cn (G.W.)

**Keywords:** radionuclide, biomaterials, targeted delivery, theranostics, combination therapy, clinical translation

## Abstract

This paper presents a comprehensive review of recent advancements in radionuclide-labeled biomaterials for cancer therapy, with a particular focus on the characteristics, production methods, and labeling techniques of α-particle, β-particle, and Auger electron-based radiotherapy. It explores innovative strategies for targeted delivery systems and highlights the advantages of theranostics and combination therapies. The application of radionuclide-labeled biomaterials in various cancer types, including prostate cancer, breast cancer, neuroendocrine tumors, gliomas, and melanoma, is systematically summarized. Furthermore, the article critically examines current technological bottlenecks and challenges in clinical translation, while proposing future directions such as AI-assisted dose optimization and multimodal combination therapies. This review provides essential theoretical foundations and practical insights to facilitate the clinical translation of radionuclide-labeled biomaterials.

## 1. Introduction

Targeted radionuclide therapy (TRT) represents a pivotal branch of precision oncology, utilizing ionizing radiation (α/β particles or Auger electrons) emitted from radionuclide decay to directly damage tumor cell DNA, thereby achieving effective tumor eradication [1]. Central to this approach is the targeted delivery system that precisely concentrates radionuclides within the tumor microenvironment, generating localized high-dose radiation while minimizing damage to normal tissues. Compared to conventional radiotherapy, TRT offers distinct advantages: (1) Enhanced targeting specificity: Utilizing various delivery vehicles (antibodies, peptide receptors, small molecules, nanoparticles, and microspheres) enables precise radionuclide accumulation in tumor microenvironments, significantly reducing off-target effects [2,3,4,5,6,7,8,9,10,11,12,13,14,15,16,17,18,19]. (2) Localized high-dose delivery: Alpha particles’ short range (50–100 μm) and high linear energy transfer (LET, ≈100 keV/μm) facilitate intensive tumor cell killing [20]. (3) Overcoming drug resistance: Radiation-induced DNA double-strand breaks bypass common chemotherapy resistance mechanisms, offering new therapeutic possibilities for refractory tumors.

However, targeted radionuclide therapy still faces multiple challenges in practical applications. First, the intrinsic pharmacokinetic limitations of radionuclides—such as rapid clearance and off-target toxicity—restrict their therapeutic efficacy. Second, the inadequate targeting specificity and stability of traditional radiopharmaceuticals result in low tumor uptake and high non-target organ toxicity. Finally, bottlenecks in radionuclide supply (e.g., ^225^Ac) and large-scale production further hinder their clinical translation. In recent years, advancements in biomaterial carriers have provided critical solutions to enhance the stability, targeting efficiency, and safety of radionuclides. The development of novel nanocarriers (e.g., gold nanoparticles and mesoporous silica) and multifunctional targeting molecules (e.g., bispecific antibodies and heterodimeric peptides) has significantly improved radionuclide enrichment in tumors and enabled multimodal therapeutic strategies, such as theranostics and radiotherapy–immunotherapy combinations [10,14,17,21]. Notably, these biomaterial innovations systematically incorporate bionic design principles, demonstrating a conscious emulation of biological systems. Antibody-mediated targeting reflects immune recognition mechanisms, while enzyme-responsive designs leverage lysosomal metabolic pathways, as demonstrated in the enzyme-responsive nanocarriers for anticancer drug delivery [22].

This review systematically evaluates the technological advancements in radionuclide-labeled biomaterials from 2015 to 2024, focusing on the following aspects: (1) the properties, production methods, and labeling techniques of therapeutic radionuclides; (2) innovative strategies for targeted delivery systems; (3) the advantages of theranostics and combination therapies; (4) clinical applications and case analyses; and (5) challenges and future directions. By integrating foundational research and clinical data, this review provides essential theoretical and practical insights into the clinical application of radionuclide-labeled biomaterials. Furthermore, it outlines future research directions to promote their broader utilization in precision oncology.

## 2. Common Types of Radionuclides Used in Therapy

### 2.1. Beta Particle

The key radionuclides for beta-particle therapy are ^177^Lu and ^90^Y, whose longer range (2–12 mm) is well-suited for treating large tumor volumes. Additionally, their crossfire effect helps overcome tumor heterogeneity.

#### 2.1.1. Lutetium-177

Lu-177 is a beta-particle emitter that decays into the stable isotope Hafnium-177 (^177^Hf, t_1/2_ = 6.64 days) [23]. The maximum beta energy of this decay is 496.8 keV (79.4%), while the average beta energy is only 133.6 keV. The energy of beta particles determines their tissue penetration range, which varies accordingly [23]. For ^177^Lu, the maximum penetration range is 2.2 mm, and the average range is 0.67 mm. In addition to beta particles, low-energy gamma rays are emitted during the decay process (e.g., 112.9 keV (6.17%) and 208.4 keV (10.4%)). This renders it appropriate for conducting in vivo biodistribution imaging by means of gamma cameras [24].

Currently, there are two methods for producing ^177^Lu: the carrier-added method and the no-carrier-added method [25]. These involve irradiating targets of either ^176^Lu (direct route) or ^176^Yb (indirect route) with neutrons in a nuclear reactor to obtain ^177^Lu [26]. The carrier-added method uses stable lutetium (^176^Lu) as the neutron irradiation material. The advantages of this method include high nuclear reaction probability, high yield, and straightforward radiochemical processing, limited to producing lutetium chloride. However, because only ^177^Lu decays and it cannot be completely separated from ^176^Lu, the specific activity of the radionuclide is reduced. Additionally, the production of the radionuclide impurity ^177m^Lu during irradiation, which has a long half-life, may complicate radiation protection and waste management [26]. The alternative method involves irradiating ^176^Yb to form ^177^Yb (t_1/2_ = 1.92 h), which will decay to become ^177^Lu. This allows for chemical separation, but the downside is the need for a significant amount of enriched ^176^Yb (1 g), leading to two main challenges. First, ^177^Lu must be efficiently separated from the target material. Second, ^176^Yb is currently only available from low-throughput facilities in Russia [26].

For the labeling of ^177^Lu, the most commonly used bifunctional chelators include DOTA, DTPA, and NOTA, among others [27]. Taking DOTA as an example, the labeling conditions involve using 0.5 M ascorbic acid as a buffer, adjusting the pH of the mixture to 4.5, and maintaining the temperature at 80–90 °C for a reaction time of 30 min. When labeling TATE, the radiochemical yield (RCY) exceeds 98% [28]; for PSMA-617, the RCY surpasses 95% [25].

#### 2.1.2. Yttrium-90

^90^Y is a pure beta-emitting radionuclide with a half-life of 64.0 h. It is characterized by high-energy emission (E_β-_avg = 934 keV, 100%) and a relatively long soft tissue range (11 mm) [29,30]. Through beta decay, ^90^Y emits a beta particle and an antineutrino, transforming into zirconium-90 (^90^Zr). In this process, a neutron in the ^90^Y nucleus converts into a proton, accompanied by the release of a high-energy beta particle with a maximum energy of 2.27 MeV and an average energy of 0.9336 MeV [31]. In 2002, Zevalin (^90^Y-tiuxetan-ibritumomab) was approved by the U.S. Food and Drug Administration (FDA) for radioimmunotherapy in the treatment of non-Hodgkin’s lymphoma. Additionally, yttrium-90-containing microspheres (SIR-spheres and TheraSphere) have also received FDA approval for use in brachytherapy for hepatocellular carcinoma [32].

Despite the proven clinical utility of ^90^Y, its widespread adoption has been hindered by the limited availability of the radionuclide due to the absence of commercially viable generator systems [33]. ^90^Y can be directly produced through the neutron capture reaction (n, γ), where neutrons (n) bombard ^89^Y (yttrium-89). However, this method faces challenges due to the low reaction cross-section (1 millibarn) and the identical chemical properties of ^80^Y and ^90^Y, making the separation of ^89^/^90^Y difficult. Consequently, this approach results in a low specific activity of the produced ^90^Y [34].

^90^Y is commonly labeled using chelators such as DOTA or CHX-A″-DTPA to form stable Y^3+^ complexes [32]. DOTA is the preferred chelator for labeling ^90^Y, with optimal labeling conditions including 0.4 M ammonium acetate (NH_4_OAc) as the buffer, pH adjusted to 5, and reaction at 90 °C for 25 min [35,36]. While DTPA (diethylenetriamine pentaacetic acid) can also effectively radiolabel ^90^Y, its rigid structure is advantageous for minimizing metal release [32]. Additionally, derivatives of DOTA, such as p-SCN-Bn-DOTA, p-SCN-Bn-DTPA, p-SCN-Bn-CHX-A′′-DTPA, p-SCN-Bn-NOTA and p-SCN-Bn-PCTA, have been explored for ^90^Y labeling, though their performance is generally less effective compared to DOTA [32]. These findings highlight the critical role of chelator design in achieving efficient and stable radiolabeling for therapeutic applications.

#### 2.1.3. Iodine-131

^131^I is a strong gamma (γ^−^) emitter with a half-life of 8.02 days and is widely utilized in beta (β^−^) therapy due to its β^−^ decay mode [37]. It emits gamma rays with maximum energies of 364 keV (89% abundance) and 606 keV (81% abundance), as well as beta rays, making it a versatile radionuclide for both imaging and therapeutic purposes [38]. The final decay product of ^131^I is the relatively stable ^131^Xe [39]. The gamma rays can be detected by gamma cameras or SPECT (Single-Photon Emission Computed Tomography) to provide imaging information, while the beta rays cause significant damage to tumor cells through radiolysis, making ^131^I suitable for intratumoral radionuclide therapy in nuclear medicine [40].^131^I was first introduced into clinical practice on 1 January 1941, by Saul Hertz for the treatment of thyroid diseases. Since then, it has become a cornerstone in the management of thyroid disorders, including hyperthyroidism and thyroid cancer. Beyond thyroid applications, ^131^I is also used for the diagnosis and treatment of neuroblastoma, pheochromocytoma, and paraganglioma [37]. Its dual role in imaging and therapy underscores its importance in nuclear medicine, offering both diagnostic precision and effective therapeutic outcomes.

The majority of ^131^I is produced through neutron irradiation of ^130^Te (tellurium-130). During this process, ^130^Te captures neutrons to form ^131^Te (tellurium-131), which undergoes beta (β^−^) decay to produce ^131^I (iodine-131) with an efficiency of 95.5% [41]. This method is widely utilized due to its high yield and reliability in generating ^131^I for medical and industrial applications. An alternative, though less commonly used, method for producing or recovering ^131^I involves uranium fission. In this process, highly enriched uranium is irradiated in research reactors, where the fission reaction of ^235^U (uranium-235) occurs, denoted as ^235^U (n, f) ^99^Mo (molybdenum-99). However, this reaction also generates a series of other isotopes, such as ^133^Xe (xenon-133), with ^131^I accounting for only about 3% of the total fission products [42,43]. This low yield makes the uranium fission method less efficient for ^131^I production compared to the neutron irradiation of ^130^Te. These production methods highlight the complex processes involved in obtaining ^131^I, a critical radionuclide in nuclear medicine for both diagnostics and therapy. The neutron irradiation of ^130^Te remains the preferred approach due to its higher efficiency and practicality.

Labeling compounds or biomaterials with radioactive iodine can be achieved through several methods, including substitution reactions, prosthetic group modification, transition metal complexation, isotope exchange, radiochemical doping, and adsorption. The primary techniques for iodine labeling focus on substitution reactions, prosthetic group modification, and transition metal complexation [40]. Among these, substitution reactions are the most extensively employed for labeling small molecules, antibodies, proteins, and specific residues such as tyrosine, histidine, or phenol derivatives. This method relies on the exchange of radioactive iodine with hydrogen atoms in the target molecules [44].

### 2.2. Alpha Particle

**Figure 1:**
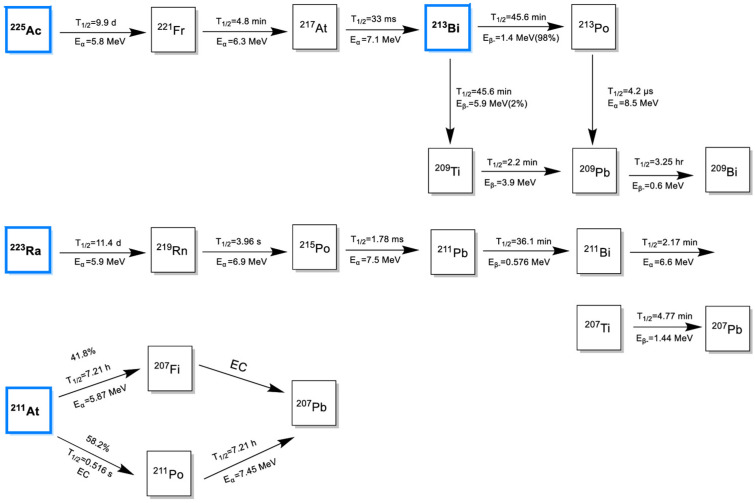
Decay scheme of some α-emitters.

Alpha particle-emitting radionuclides, such as actinium-225, astatine-211, and bismuth-213, are at the forefront of targeted cancer therapy due to their unique physical properties (Figure 1). These radionuclides emit alpha particles, which are characterized by a short penetration range (50–100 μm) and a high linear energy transfer (LET) of 80–230 keV/μm [20]. These attributes enable α-particles to deposit a significant amount of energy within a highly localized area, ensuring precise and effective destruction of tumor cells while minimizing damage to surrounding healthy tissues [20].

#### 2.2.1. Radium-223

^223^Ra is an alpha-emitting radionuclide with a half-life of 11.4 days. It demonstrates a unique ability to selectively target areas of increased bone turnover, such as those found in bone metastases. The high-energy alpha particles emitted by ^223^Ra have a short penetration range (<100 μm), making them highly effective in delivering localized radiation with minimal damage to the surrounding healthy tissue [45]. The decay chain of ^223^Ra involves five daughter nuclides, ultimately reaching the near-stable isotope lead-207 (^207^Pb) as shown in Figure 1. During its decay process, ^223^Ra and its progeny emit alpha particles (accounting for 95.3% of the total energy), beta particles (3.6%), and gamma radiation (1.1%) [46]. Historically, ^223^Ra was obtained from uranium mill tailings containing pitchblende ores. However, the modern clinical and commercial production of ^223^Ra is achieved using actinium-227 (^227^Ac) as a long-term generator. Actinium-227, with a half-life of 21.8 years, primarily undergoes beta decay to thorium-227 (^227^Th), serving as a sustained source for ^223^Ra production. The process begins with neutron irradiation of natural radium-226 (^226^Ra) to produce actinium-227, which is then immobilized on an Ac-resin for the purification of ^223^Ra [47].

To harness the therapeutic potential of ^223^Ra, this alpha-emitting radionuclide must be stably conjugated to tumor-targeting vectors using bifunctional chelators (BFCs). However, identifying an ideal chelator for ^223^Ra remains a significant challenge. Macropa, an expanded 18-membered macrocyclic ligand featuring two pendant picolinate donor arms, has emerged as a highly effective chelator for ^223^Ra. It exhibits excellent radiolabeling efficiency and long-term stability, making it a promising candidate for clinical applications. Radiolabeling with Macropa is conducted at room temperature using a metal-free ammonium acetate buffer (0.1 M, pH 6), with a reaction time of just 5 min [48].

#### 2.2.2. Astatine-211

^211^At is an alpha-emitting radionuclide with a relatively short half-life (t_1/2_ = 7.21 h). Despite its brief lifespan, the half-life of ^211^At is sufficient to support multi-step radiolabeling processes, making it compatible with a variety of targeting vectors, including small molecules, peptides, and antibodies/fragments [49]. Similar to other targeted alpha therapy (TAT) candidates, the LET of ^211^At is quite high, reaching approximately 100 keVμm^−1^, contributing to its significant relative biological effectiveness (RBE) [50]. The ionization density of ^211^At is comparable to the diameter of the DNA double helix, enabling it to generate more than 10 ionizations within a cylindrical volume of 100 Å in diameter and 3 nm in length. ^211^At is considered a pure alpha emitter, with 100% of its energy released as two alpha particles with energies of 5.87 MeV and 7.45 MeV (Figure 1) [51,52].

There are two primary methods for producing ^211^At, each with its unique advantages and challenges [53]. The most commonly used method involves the irradiation of bismuth-209 (^209^Bi) with alpha particles in a cyclotron. The nuclear reaction can be represented as follows: ^209^Bi (α, 2n) ^211^At. In this process, alpha particles interact with bismuth nuclei to produce ^211^At, accompanied by the release of two neutrons. A critical consideration is the control of alpha beam energy, which must be carefully maintained below 29.0 MeV (typically 28.0–29.5 MeV) to minimize the production of astatine-210 (^210^At) through the competing reaction ^209^Bi (α, 3n) ^210^At, which becomes significant at energies above 30 MeV [53,54]. An alternative method involves placing suitable target materials in a nuclear reactor and utilizing the neutron flux for irradiation. While this approach can theoretically produce ^211^At, its application is less widespread due to the complexity of the nuclear reactions involved and the challenges associated with separating the desired product [53].

^211^At can be labeled using two primary methods [55], each tailored to specific applications and chemical frameworks. The most widely used approach involves organometallic compounds, such as N-succinimidyl 3-(trialkylstannyl) benzoate and related compounds (e.g., compound **1**), which facilitate electrophilic substitution reactions. A notable advancement in this area is the one-step labeling method developed by the Gothenburg team, where trialkylstannyl benzoate is pre-coupled before radiolabeling [56]. Another approach involves nucleophilic substitution in which the unactivated arylboronic acid derivative reacts rapidly with an electrophilic astatine species [55]. In addition to the above two labeling methods, aryl iodinium salts and isothiocyanatophenyl-closo-decaborate (2-) (B10) boron cage molecules can be used for mAb conjugation labeling [57].

#### 2.2.3. Bismuth-213

^213^Bi is another short-lived α-emitting radionuclide, with a half-life of only 45.6 min [58]. Due to its unique properties, ^213^Bi has shown significant promise in the treatment of neuroendocrine tumors when conjugated with low-molecular-weight, rapidly diffusing peptides such as DOTATOC and Substance P analogues [59,60]. The decay pathway of ^213^Bi is illustrated in Figure 1. Notably, its decay ultimately results in the stable isotope ^209^Bi, ensuring minimal long-term radiological impact.

^213^Bi originates from the decay chain of ^225^Ac and is clinically produced using a “direct”-working ^225^Ac/^213^Bi generator. In this setup, ^225^Ac is adsorbed onto the cation-exchange resin AG MP-50, while ^213^Bi is eluted for therapeutic use [61]. A standard ^225^Ac/^213^Bi generator retains ^225^Ac on 0.33 mL of AG MP-50 resin, with an initial activity of 1.1–1.3 GBq. The elution of ^213^Bi is achieved using 0.6 mL of a 0.1 M NaI/0.1 M HCl solution, yielding an elution efficiency of 76 ± 3%. Importantly, the breakthrough of ^225^Ac is minimized to less than 2 × 10^−5^%, ensuring high purity and safety [62].

DOTA and CHX-A″-DTPA are commonly used for labeling ^213^Bi; however, neither chelator exhibits optimal compatibility with Bi^3+^ [55]. Both DOTA and CHX-A″-DTPA complexes demonstrate limited stability in human plasma (85% for DOTA and 76% for CHX-A″-DTPA after 2 h) and require stringent labeling conditions (95 °C or 80 °C for 5 min) [63]. To address these limitations, researchers have developed alternative chelators, such as LPy and DOTP [55]. These chelators offer milder labeling conditions. For instance, LPy can be labeled at room temperature using NH_4_OAc as a buffer at pH 5.0 [64]. DOTP requires only 5 min for labeling under conditions otherwise identical to those used for LPy [63]. ^213^Bi-DOTP showed superior stability in human serum compared to ^213^Bi-DOTA or ^213^Bi-CHX-A″-DTPA after 120 min, and it primarily accumulates in bones, unlike free Bi^3+^, which is primarily retained in the kidneys [63].

### 2.3. Auger Electron

#### Iodine-125

^125^I, with a half-life of 59.4 days, emits X-rays, γ-rays, and Auger electrons during its decay process. The relatively long half-life of ^125^I offers significant advantages in terms of transportation to distant reactor production sites and long-term storage. Due to its low dose rate, high relative biological effectiveness, and optimal therapeutic radius, ^125^I is widely used in the treatment of various malignancies, demonstrating notable advantages in local tumor control and patient survival rates [65,66]. ^125^I has become the radionuclide of choice for the production of brachytherapy seeds for the treatment of prostate and eye cancers [67,68,69,70,71].

The production of ^125^I primarily occurs through the neutron capture process of xenon. Only ^125^Xe can directly decay to ^125^I, with two primary decay pathways: (^124^Xe(n, γ)^125^Xe (57 s)→^125^I) or (^124^Xe(n, γ)^125^ Xe (19.9 h)→^125^I). However, this method presents several challenges: the technical requirements for handling gaseous targets in reactor irradiation are demanding, the process is operationally complex, and the use of natural xenon targets can produce radioactive impurities (e.g., ^126^I). In 2012, Joshi et al. successfully developed a method for producing ^125^I by neutron irradiation of natural xenon gas combined with wet distillation. This approach demonstrated excellent performance metrics and has been implemented in localized production, providing significant support to the field [72].

The labeling methods for ^125^I are the same as those for ^131^I.

## 3. Emerging Radionuclides

### 3.1. Terbium-161

^161^Tb has emerged as a promising radionuclide for therapeutic applications, characterized by a half-life of 6.9 days and the emission of low-energy β^−^ particles with an average energy of 0.15 MeV [73]. It decays into stable dysprosium-161 (^161^Dy). The physical properties of ^161^Tb closely resemble those of ^177^Lu, a clinically established radionuclide, with comparable half-lives (^177^Lu: 6.7 days) and β^−^ emission energies (^177^Lu: 0.14 MeV). Importantly, ^161^Tb emits a small number of photons with an energy of 75 keV, making it suitable for gamma camera imaging. Additionally, ^161^Tb emits Auger electrons, which exhibit a higher linear energy transfer (LET) compared to β^−^ particles. This property enhances its effectiveness in targeting small lesions, offering a potential advantage over ^177^Lu in precision therapy [73]. The therapeutic potential of ^161^Tb has been explored in tumor-targeted radionuclide therapy, with recent clinical trials demonstrating its application in prostate-specific membrane antigen (PSMA)-targeted therapy. The use of ^161^Tb-PSMA in human studies has yielded promising results, with successful SPECT/CT imaging and favorable therapeutic outcomes [74].

^161^Tb is produced through neutron irradiation of gadolinium-160 (^160^Gd) with a thermal neutron capture cross-section of 1.5 barns. This process involves the nuclear reaction ^160^Gd (n, γ)^161^Gd, followed by the β^−^ decay of the short-lived intermediate ^161^Gd to yield ^161^Tb. After production, ^161^Tb can be separated from the target material using chromatographic techniques, such as the separation of dysprosium (Dy) and terbium (Tb) [44,46]. However, the low loading capacity of this method poses challenges for large-scale production [73,75].

The radiolabeling of ^161^Tb is analogous to that of lutetium-177 (^177^Lu), commonly employing bifunctional chelators such as DOTA. The radiolabeling process typically involves mixing ^161^Tb with the chelator in a 0.5 M sodium acetate buffer, adjusting the pH to 4.5–5.0, and heating the mixture at 90 °C for 15 min. This procedure achieves a radiochemical yield (RCY) of over 98% and ensures radiolabeling stability comparable to that observed with ^177^Lu [76,77].

### 3.2. Actinium-225

^225^Ac, which has a relatively long half-life (t_1/2_ = 9.9 days), decays through a series of six daughter nuclides, culminating in the nearly stable bismuth-209 (^209^Bi) (half-life: ~19 × 10^18^ years) [78]. The decay chain of ^225^Ac begins with the emission of an α-particle (E_α_ = 6.3 MeV), resulting in the formation of francium-221 (^221^Fr) (t_1/2_ = 4.8 min). This is followed by the decay to astatine-217 (^217^At) (E_α_ = 7.1 MeV, t_1/2_ = 33 ms) and subsequently to bismuth-213 (^213^Bi) (E_α_ = 5.8 MeV, t_1/2_ = 45.6 min). ^213^Bi primarily undergoes β decay (98%, E_β_ = 1.4 MeV) to polonium-213 (^213^Po), with a minor branch (2%) undergoing α decay (E_α_ = 5.9 MeV) to thallium-209 (^209^Tl). Both ^213^Po (E_α_ = 8.4 MeV, t_1/2_ = 3.7 μs) and ^209^Tl decay to lead-209 (^209^Pb) (E_β_ = 1.4 MeV), which ultimately stabilizes as ^209^Bi (Figure 1) [79]. The sequential emission of four α particles and two β particles within a relatively short timeframe generates multiple daughter isotopes, significantly enhancing the therapeutic potential of ^225^Ac-labeled compounds [80].

There are five primary methods for producing ^225^Ac [81]. The first method involves the separation of ^225^Ac from its parent isotope, ^229^Th. This process includes dissolving ^229^Th in 8 M nitric acid, followed by the separation of ^225^Ac and ^225^Ra using an anion exchange resin. Discrete ^225^Ac is then obtained through advanced techniques such as cation exchange column chromatography, solid-phase extraction chromatography, or the synergistic application of anion and cation exchange resin systems [82,83]. The second method utilizes a cyclotron to irradiate natural ^232^Th targets with medium-to-high-energy protons [20]. The third method employs an electron accelerator, where a tungsten target is irradiated with an incident electron beam to generate bremsstrahlung photons [84]. The fourth method involves the use of a proton accelerator to produce ^225^Ac. Specifically, ^226^Ra targets are irradiated with protons in a cyclotron, and ^225^Ac is generated through the ^226^Ra (p, 2n) ^225^Ac reaction [20]. This method is considered the most viable for large-scale production of ^225^Ac. The fifth method relies on nuclear reactors to produce ^225^Ac, where ^225^Ra is generated via the ^226^Ra (n, 2n) ^225^Ra reaction using high-energy neutrons (>6.4 MeV). These neutrons are moderated by the highly toxic isotope ^227^Ac (t_1/2_ = 21.8 years) [85].

^225^Ac is typically labeled using chelating agents to ensure efficient and stable binding. Davis et al. [86] conducted a pivotal study in 1999, comparing ^225^Ac-acetate labeling with several radiometal chelators, including EDTA, PEPA, and CHX-DTPA. The study revealed that the radiochemical yields of EDTA, PEPA, and CHX-PEPA reached 80–90% in 1 M ammonium hydroxide (NH_4_OH). Notably, CHX-DTPA and PEPA conjugates exhibited reduced hepatic uptake, indicating superior in vivo stability. In the same year, Deal et al. [87] introduced HEHA for ^225^Ac complexation. The HEHA-monoclonal antibody (mAb) conjugates demonstrated labeling yields of 60–85% in 0.15 M ammonium acetate (NH_4_OAc), with specific activities ranging from 7.4 × 10^6^ to 1.48 × 10^7^ Bq/mg of protein. Subsequently, McDevitt et al. [88] investigated the radiolabeling efficiency and stability of several chelators, including DTPA, DOTA, TETA, DOTMP, and related derivatives. Among these, DOTA emerged as the most effective, achieving optimal radiolabeling in 3 M ammonium acetate and maintaining high stability in 25% serum samples. After 10 days, 90% of the DOTA-^225^Ac complex remained intact, underscoring its robustness for in vivo applications.

The basic properties of the radionuclides are shown in (Table 1).

## 4. Innovation in Targeted Delivery Systems

Radionuclide-labeled biomaterial-based targeted delivery systems are central to precision cancer therapy. Their design must balance targeting specificity, drug-loading capacity, and biosafety. This section systematically reviews recent technological breakthroughs and challenges in antibody and peptide receptor targeting, small molecules, nanomaterials, microspheres, aptamers, biological carriers and bone cements.

### 4.1. Antibodies and Peptides

Antibodies are widely used in radionuclide delivery systems due to their high affinity and specificity [2,3,4,5,6,7,8]. Monoclonal antibodies (mAbs) enable precise delivery of radionuclides by targeting overexpressed antigens on tumor surfaces. Nevertheless, due to their substantial molecular weight (~150 kDa), these molecules exhibit limited penetration into tumor tissues and extended circulation in the bloodstream, potentially elevating off-target organ toxicity [89]. Recent advancements in antibody engineering have significantly improved targeting efficiency and tumor penetration. Puttemans et al. [2] developed an anti-HER2 single-domain antibody (2Rs15d) conjugated with various radionuclides (e.g., ^111^In, ^225^Ac, and ^131^I), which significantly extended median survival in two tumor models that were less responsive to trastuzumab monotherapy. To address the limitations of intact antibodies, pepsin digestion can be used to cleave intact antibodies into bivalent F(ab′)2 fragments. Radiolabeled ^177^Lu-CHX-A″-DTPA-F(ab′)2-Trastuzumab exhibits cytotoxicity comparable to that of traditional monoclonal antibodies [3].

Short peptide molecules have gained prominence as versatile platforms for targeted drug delivery, owing to their minimal immunogenic potential and high synthetic adaptability. Somatostatin analogs (SSAs) serve as clinically established ligands for peptide receptor radionuclide therapy (PRRT), particularly in the management of neuroendocrine tumors. For instance, ^177^Lu-DOTATOC has gained FDA approval, but its efficacy is limited by receptor subtype heterogeneity (e.g., better response in SSTR2-overexpressing tumors). Recent studies propose an “agonist-antagonist hybrid strategy,” where antagonists enhance tumor retention time, significantly improving the uptake of ^177^Lu-labeled drugs in tumors [9]. Dual-targeting short peptides enhance radionuclide accumulation by simultaneously targeting multiple tumor-associated antigens, offering a novel approach for radionuclide delivery. For example, the heterodimeric peptide iRGD-C_6_-lys(^211^At-ATE)-C_6_-DA7R demonstrates dual targeting capability against VEGFR and integrin α_v_β_3_, achieving a tumor-to-background ratio of 12:1 for ^211^At-labeled drugs without significant hepatotoxicity or nephrotoxicity [10].

### 4.2. Small Molecules

Small-molecule delivery systems have demonstrated unique advantages in tumor diagnosis and therapy. Their small size enables efficient penetration into tumor tissues, overcoming the heterogeneous distribution issues associated with macromolecules and nanocarriers. Additionally, their simple structures facilitate facile synthesis and modification, allowing for rapid optimization of pharmacokinetic properties. Moreover, small molecules exhibit rapid metabolism, reducing radionuclide accumulation in normal tissues and minimizing adverse effects. Their low immunogenicity further enhances safety. For example, ^18^F-FDG and ^68^Ga-PSMA-11, which target tumor metabolic pathways or cell surface receptors, have shown outstanding performance in tumor diagnosis [11,12], while ^177^Lu-NM600 exhibits therapeutic potential for TNBC due to its excellent tumor-targeting properties [13].

However, small-molecule delivery systems also have limitations. Some small molecules exhibit limited targeting specificity, which may lead to non-specific accumulation of radionuclides in non-tumor tissues, as observed with ^68^Ga-FAPI-04 in certain benign lesions [90]. Additionally, small molecules lack sustained release capabilities, unlike microspheres or nanoparticles, necessitating precise control of therapeutic dosages. Future research directions include the development of novel small-molecule probes, optimization of radionuclide labeling techniques, and combination with other delivery systems to further enhance their efficacy and application scope.

### 4.3. Nanocarriers

Nanocarriers are nanoscale materials designed to deliver drugs, genes, or other therapeutic agents to targeted biological locations with high specificity. They are typically composed of biocompatible materials and can be categorized into inorganic nanoparticles and organic nanoparticles. Nanocarriers generally range in size from 1 to 100 nanometers, enabling them to efficiently traverse biological barriers (e.g., blood vessel walls) and target specific cells or tissues [91].

Inorganic nanoparticles are nanoscale structures composed of inorganic materials such as metals, metalloids, non-metals, carbon nanomaterials, and semiconductor quantum dots, with at least one dimension smaller than 1000 nanometers. They exhibit high loading capacity, excellent biocompatibility, prolonged circulation time in the bloodstream, and controllable release of active agents [91]. Gold nanoparticles (AuNPs), owing to their high atomic number and ease of functionalization, serve as ideal carriers for the α-emitter ^225^Ac. Studies have shown that intratumoral injection of ^225^Ac-labeled AuNPs (Au@TADOTAGA) delays glioma progression through localized high-dose radiation, achieving 72% tumor volume inhibition with a single injection and less than 5% escape rate of the daughter nuclide ^213^Bi [14]. Silica-based core-shell structures (C’ dots), when surface-modified with melanoma-targeting MC1-R peptides, demonstrate significantly improved pharmacokinetics: C’ dots with sizes below 10 nm enable renal clearance, reduce hepatic accumulation, and enhance tumor uptake by 4.8-fold compared to free peptides [15]. Porous iron oxide nanoparticles (PIONs), widely used as versatile magnetic nanoplatforms in drug delivery, form concentrated colloidal nanoclusters that enable direct radiolabeling with ^68^Ga and ^177^Lu for PET imaging and cancer therapy [16]. Additionally, PIONs are applicable in magnetic resonance imaging (MRI), photoacoustic imaging (PAI), and photothermal therapy, holding future potential for novel combinations of radionuclide therapy with these diagnostic and therapeutic modalities [16,92].

Organic nanocarriers are nanoscale structures composed of organic materials, with structural features below 1 μm in at least one dimension. These architectures demonstrate remarkable versatility in incorporating therapeutic agents through encapsulation, binding, or loading active components. Their distinctive advantages include superior biocompatibility, tunable biodegradation profiles, substantial payload capacity, balanced amphiphilic character, and minimal cytotoxic effects [91]. The porous structure of mesoporous silica (PDA-mSiO_2_) enables the co-delivery of chemotherapeutic drugs and radionuclides. For example, nanoparticles co-loaded with doxorubicin and ^177^Lu demonstrated a chemotherapy–radiotherapy synergistic effect in a triple-negative breast cancer (TNBC) model: following DNA damage induced by doxorubicin, the β-rays from ^177^Lu further triggered oxidative stress, increasing the complete tumor remission rate to 65% [17]. Liposome systems, through surface modifications such as targeting glucose transporters, enhance tumor accumulation. Radioactively labeled glucose-modified liposomes (^177^Lu-GML) achieved a tumor uptake of 5.8% ID/g in a colorectal cancer model, with 80% retention over 72 h [18].

### 4.4. Microspheres

Microsphere delivery systems demonstrate unique advantages in tumor-targeted therapy. Their larger particle size and controllable drug-loading capacity enable localized distribution and sustained release of radionuclides, thereby delivering high-dose radiation to specific sites (e.g., liver tumors) while minimizing damage to surrounding normal tissues [93]. Additionally, microspheres can enhance therapeutic efficacy by embolizing tumor blood supply. For instance, ^90^Y microsphere radioembolization has been widely employed in the radiotherapy of liver cancer and, when combined with immunotherapy (e.g., durvalumab), significantly prolongs patient survival [19]. Furthermore, the high stability of microspheres allows for prolonged in vivo release of radionuclides, offering potential for long-term treatment.

However, microsphere delivery systems also exhibit certain limitations. Their relatively large particle size limits penetration into tumor tissues, restricting their application in non-vascular tumors. Additionally, the preparation process for microspheres is complex and costly, and their non-degradable nature poses potential risks of long-term retention in the body. Moreover, uneven dose distribution may lead to insufficient radiation in certain tumor regions, compromising therapeutic efficacy. Future research directions include optimizing the size and degradation properties of microspheres, developing novel radionuclide labeling techniques, and exploring combined applications with other delivery systems (e.g., small molecules or nanoparticles) to further enhance targeting capability and therapeutic outcomes.

Bioactive glass microspheres represent a revolutionary advancement in selective internal radiation therapy (SIRT), particularly for unresectable hepatocellular carcinoma. In contrast to conventional non-bioactive glasses, these materials form stable interfaces with host tissues without inducing fibrosis, while their chemical composition (e.g., Y_2_O_3_-Al_2_O_3_-SiO_2_) can be precisely tailored to control degradation rates and radionuclide binding kinetics [94]. The incorporation of β-emitting radionuclides (e.g., ^90^Y, ^32^P) into the glass matrix during fabrication ensures structural immobilization, minimizing post-activation leakage risks [95,96].

Two FDA-approved ^90^Y-loaded microspheres dominate clinical SIRT: TheraSphere^®^ (yttrium aluminosilicate glass) and SIR-Spheres^®^ (resin-based). TheraSphere^®^ microspheres (20–30 μm diameter) exhibit superior chemical durability due to yttrium’s integration into the glass lattice, whereas SIR-Spheres^®^ rely on ion exchange resin coatings [31,96]. Both achieve comparable tumoricidal efficacy through hepatic arterial delivery, leveraging the tumor’s preferential arterial vascularization [97,98]. Phosphate-based glass variants (e.g., 42P_2_O_5_-12Al_2_O_3_-4SiO_2_-44MgO) offer resorbable alternatives with degradation rates tunable from days to years, addressing persistent foreign body concerns of non-degradable silicates [99,100].

### 4.5. Aptamers

Aptamers represent a class of single-stranded oligonucleotides (DNA, RNA, or chemically modified variants) that demonstrate high-affinity, specific binding to molecular targets while exhibiting minimal immunogenicity. These synthetic ligands are typically selected through Systematic Evolution of Ligands by Exponential Enrichment (SELEX), enabling the identification of sequences with dissociation constants (Kd) in the nanomolar to picomolar range for targets ranging from small molecules to transmembrane receptors [101,102]. Anaplastic thyroid cancer (ATC) is an exceptionally aggressive and poorly differentiated form of thyroid cancer that exhibits strong resistance to standard treatments such as surgical resection and radioactive iodine therapy. Aptamer AP-1-M, targeting CD133, which is highly expressed on ATC cells, has been developed and, when conjugated with doxorubicin, significantly inhibits tumor cell proliferation [103]. Aptamers offer distinct advantages over antibodies, including smaller size (8–25 kDa vs. 150 kDa for IgG), easier chemical synthesis, and reversible thermal denaturation for storage stability [104]. One of the most compelling aspects of aptamer-based technology is its ability to bind to nanoparticles, enabling controllable and selective anti-tumor effects, especially when utilizing stimulus-responsive nanoparticles [105,106]. González-Ruíz et al. developed a ^177^Lu-Au-NLS-RGD-Aptamer system with fluorescent and anti-angiogenic properties. This system can target vascular endothelial growth factor (VEGF) through the VEGF-specific aptamer, and α_v_β_3_ integrin via the NLS-RGD (nuclear localization sequence-Arg-Gly-Asp) peptide [107].

### 4.6. Biological Carriers

Biological carriers such as bacteria, cells, viruses, and their biological derivatives (e.g., extracellular vesicles, EVs) possess unique attributes, including the ability to synthesize targeting molecules, excellent biocompatibility, and numerous binding sites. These carriers leverage intrinsic biological mechanisms for tumor targeting: bacteria utilize chemotaxis toward tumor-secreted metabolites and hypoxia-responsive proliferation; immune cells exploit tumor homing through surface receptor-ligand interactions; EVs inherit parental cell membrane proteins for organotropism; while oncolytic viruses selectively replicate in tumor cells with defective interferon pathways [108]. These features make them highly promising in enhancing radionuclide-mediated tumor therapies [109]. Radioactively labeled biological carriers can increase the accumulation of radionuclides in tumors and reduce radiation-induced damage to normal tissues. Furthermore, radionuclide-labeled biological carriers may activate immune responses or exhibit oncolytic effects, making them suitable for combination therapies [108,110]. For instance, iodine-mediated oxidation reactions using ^131^I-labeled inactivated Salmonella VNP20009 (VNP) enable the combination of radiation therapy (RT) with immunotherapy, resulting in a potent anti-tumor effect [111]. However, inactivated bacteria have limited ability to penetrate and target distant tumors. This issue can be overcome by labeling the attenuated (at) live *Listeria monocytogenes* (Listeria^at^) with ^188^Re [112]. Modified cells as biological carriers can also improve radionuclide delivery efficiency, inducing effective anti-tumor immunity. Kurtz et al. designed CD19 CAR-T cells that express the huC825 single-chain antibody fragment (scFv) on their surface, utilizing the strong binding affinity between huC825 and metalized DOTA, as well as the biological properties of T cells. These cells can bind the therapeutic radionuclide ^86^Y-aminobenzyl-DOTA, offering new opportunities for precise delivery and multiple administrations [113].

Additionally, virus-based radionuclide carriers can synergize RT with oncolytic virotherapy, producing enhanced anti-tumor effects. For example, engineered viral vaccines that infect tumor cells and express SSTR significantly improve the tumor-targeting capability of the radionuclide-labeled octreotide analogue (^177^Lu-DOTATOC) [114]. Engineered exosomes with multifunctional capabilities have emerged as promising delivery systems for tumor-specific diagnostics and treatment, garnering considerable interest in oncology research. Exosomes are nanoscale, single-layer membrane vesicles secreted by mammalian cells. By engineering HEK-293T cells to express an exosomal membrane protein (Lamp2b) fused with an αv integrin-specific iRGD peptide and a tyrosine fragment, a targeted delivery platform based on HEK-293T exosomes has been developed. These multifunctional exosomes (Dox@iRGD-Exos-^131^I) can efficiently target α_v_β_3_ integrin-positive ATC cells, delivering ^131^I and doxorubicin specifically to tumor tissues and significantly inhibiting tumor growth [115].

### 4.7. Bone Cements

Bone cements have evolved into multifunctional therapeutic platforms for spinal metastasis management, synergizing mechanical stabilization with localized radiotherapy. Polymethylmethacrylate (PMMA), the gold standard in vertebroplasty, is now engineered to incorporate β-emitting radionuclides (e.g., ^166^Ho, ^153^Sm) within calcium phosphate composites, achieving dual mechanical support and sustained radiation delivery [116,117]. The exothermic polymerization of PMMA (40–48 °C) induces tumor neovascular ablation via hyperthermia, while neutron-activated radionuclides (32.5 MBq/mg for ^166^Ho; 14.5 MBq/mg for ^153^Sm) provide localized irradiation, circumventing spinal cord exposure risks associated with conventional EBRT [118,119]. Calcium phosphate additives enhance bioactivity through ionic substitution (Ho^3+^/Sm^3+^ → Ca^2+^ in hydroxyapatite), with sol-gel-derived porosity (30–50 μm) controlling radionuclide elution kinetics [120,121]. Despite these advances, the PMMA non-degradability limits long-term biocompatibility (<0.1% annual radionuclide leaching) compared to resorbable phosphate cements (2–5% leakage), while heterogeneous thermal distribution during curing risks incomplete tumor ablation [119,121]. Future innovations focus on bioactive hybrids integrating PMMA with osteoconductive borosilicate glasses, AI-optimized 3D dosimetry models for multi-radionuclide systems, and immunomodulatory cements co-loaded with checkpoint inhibitors to amplify abscopal responses [122,123].

## 5. Advances in Theranostics

Theranostics, by integrating diagnostic and therapeutic functions, achieves the synergy of “precise localization” and “targeted eradication,” emerging as a pivotal direction in radionuclide therapy. Its core strategy involves the selection of radionuclide pairs with complementary physical properties, enabling closed-loop management of tumor staging, biodistribution verification, and therapeutic response through the same targeting molecule or carrier system [124]. This approach enhances therapeutic precision, reduces side effects, and provides a foundation for personalized treatment.

^68^Ga (t_1/2_ = 68 min) pairs well with ^177^Lu (t_1/2_ = 6.7 days) for synergistic short-term diagnosis and medium-term therapy. The PET/CT imaging agent ^68^Ga-DOTA-JR11 precisely localizes somatostatin receptor (SSTR)-positive neuroendocrine tumors (NENs), followed by targeted therapy using ^177^Lu-DOTA-JR11, which delivers β-radiation to eradicate tumors, significantly prolonging patients’ progression-free survival (PFS)[125,126].

^99m^Tc has become the predominant diagnostic radionuclide in clinical nuclear medicine practice due to its optimal nuclear characteristics, multifunctional applications, and favorable economic profile. In prostate cancer treatment, the novel radiopharmaceutical ^99^Tc^m^ (t_1/2_ = 6.02 h)/^188^Re (t_1/2_ = 16.9 h)-PSMA-GCK01, targeting prostate-specific membrane antigen (PSMA), has been successfully applied in humans for the first time, demonstrating promising preclinical evaluation results [127].

**Figure 2:**
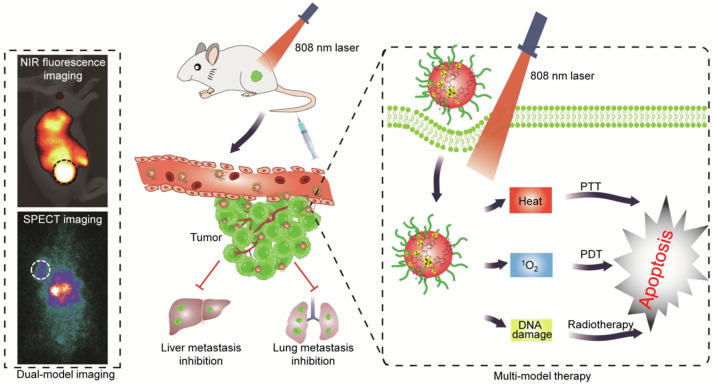
Schematic illustration of SPECT/fluorescence dual-model imaging and combinational PTT/PDT/radiotherapy of breast cancer using rSPNs [128].

^89^Zr (t_1/2_ = 3.3 days) supports multi-time-point biodistribution studies, while ^225^Ac emits high linear energy transfer (LET) α-particles with a short range, enabling efficient localized tumor eradication. For instance, after confirming targeted accumulation in melanoma using ^89^Zr-labeled submicron core-shell particles (SPs) via PET/CT, therapy with ^225^Ac-SPs significantly suppressed tumor growth and extended survival in mice. Furthermore, multimodal theranostic strategies integrating multiple imaging modalities have further enhanced diagnostic accuracy and therapeutic efficacy. For example, Yu et al. [128] developed radioactive semiconductor polymer nanoparticles (rSPNs), which combine near-infrared fluorescence and SPECT dual-modality imaging, integrating photothermal therapy (PTT), photodynamic therapy (PDT), and radiotherapy for improved anti-tumor outcomes (Figure 2).

For radionuclide pairs, the optimal choice is undoubtedly isotopes of the same element. ^133^La (half-life: 3.9 h) and ^135^La (an Auger electron emitter) perfectly meet this requirement. Researchers successfully produced ^133^La using a cyclotron and conducted preclinical PET tumor imaging studies with ^133^La-PSMA-I&T, comparing it with commonly used PET radionuclides such as ^68^Ga and ^89^Zr. The results demonstrated that ^133^La-labeled PSMA-I&T radiotracer demonstrated superior imaging performance, offering enhanced tumor delineation and spatial resolution compared to both ^68^Ga- and ^89^Zr-based imaging agents [129].

## 6. Combination Therapy Strategy

Combination Therapy Strategy with Radionuclide-Labeled Biomaterials leverages synergistic multi-mechanistic interactions to overcome the limitations of single therapeutic modalities, establishing a novel paradigm for precision oncology.

### 6.1. Radionuclide Therapy–Immunotherapy Combination

Radionuclide therapy activates anti-tumor immune responses by inducing immunogenic cell death (ICD), while immune checkpoint inhibitors (e.g., PD-1 monoclonal antibodies) enhance therapeutic efficacy by reversing T cell exhaustion [130,131]. Studies have demonstrated that ^225^Ac-labeled submicron core-shell particles (SPs) significantly inhibit tumor growth in melanoma models. The high-LET α-particle radiation triggers substantial release of damage-associated molecular patterns (DAMPs) from irradiated tumor cells. This radiation-induced immunogenic cell death stimulates dendritic cell activation and enhances tumor-infiltrating lymphocyte recruitment, effectively converting immunologically “cold” tumors into immunotherapy-responsive “hot” microenvironments [132].

In another study, the combination of ^90^Y-GZP (a radiolabeled peptide targeting granzyme B) with a PD-1 inhibitor achieved dose-dependent curative responses in a murine colon cancer model, enhancing overall survival to over 70%. The mechanism involves ^90^Y β-radiation increasing tumor antigen exposure, while the PD-1 inhibitor reverses T cell exhaustion, establishing a positive feedback loop for immune activation [133].

Additionally, to address drug resistance in metastatic castration-resistant prostate cancer (mCRPC), ^177^Lu-RIMS-1 has been developed, integrating radiotherapy and immunostimulation into a single molecular entity. This approach significantly improves therapeutic outcomes and enhances intratumoral T cell infiltration [134].

### 6.2. Radionuclide Therapy–Chemotherapy Synergy

The combination of radionuclide therapy and chemotherapeutic agents (e.g., doxorubicin, cisplatin) significantly enhances therapeutic efficacy through multi-mechanistic synergistic effects. Radionuclide radiation enhances the permeability and cytotoxicity of chemotherapeutic drugs by disrupting tumor vasculature and DNA, while chemotherapy agents amplify the radiotherapeutic effects by inhibiting DNA repair mechanisms [135]. For instance, FA-PDA@mSiO2 nanoparticles co-loaded with doxorubicin [17], surface-modified with folate targeting ligands, specifically recognize the overexpressed FRα receptors on triple-negative breast cancer (TNBC) cells. The β^−^radiation emitted by ^177^Lu induces DNA damage, while pH-responsive doxorubicin release enhances radiosensitivity by inhibiting topoisomerase II. Animal experiments demonstrated that the combination therapy increased tumor suppression by 2.3-fold compared to monotherapy and reduced lung metastases by 80%.

### 6.3. Synergistic Radiotherapy–Photothermal Therapy

In recent years, the combination of radionuclide therapy and photothermal therapy (PTT) has significantly enhanced tumor eradication by integrating high-energy radiation with localized hyperthermia effects. Huang et al. [136] developed a novel mesoporous polydopamine nanoparticle (MPDA), optimizing its morphology to enhance iodine labeling efficiency and photothermal conversion. They demonstrated that this nanoparticle enables the combination of photothermal therapy and radiotherapy, significantly improving the therapeutic outcome of adenocarcinoma. Zhao et al. designed a multifunctional nanoplatform by coating single-walled carbon nanotubes (SWNTs) with a polydopamine (PDA) shell and further modifying it with polyethylene glycol (PEG), successfully constructing the SWNT@PDA-PEG nanostructure. This platform leverages the PDA shell to achieve efficient chelation of M^2+^ and labeling of ^131^I, demonstrating excellent performance in magnetic resonance imaging (MRI) and radionuclide therapy. Furthermore, capitalizing on the exceptional near-infrared (NIR) absorption characteristics of single-walled carbon nanotubes (SWNTs), this theranostic platform combines NIR-triggered photothermal ablation with ^131^I radionuclide therapy. This synergistic approach establishes a novel combinatorial treatment paradigm for malignant tumors [137].

## 7. Applications in Tumor Therapy

### 7.1. Prostate Cancer

Prostate cancer (PCa) is a leading cause of cancer-related deaths in men worldwide. Targeted radionuclide therapy has shown significant efficacy in treating prostate cancer, especially in metastatic castration-resistant prostate cancer [138].

Due to the overexpression of prostate-specific membrane antigen (PSMA) in prostate cancer [139], β-emitting labeled ligands such as ^177^Lu-PSMA-617, ^177^Lu-PSMA I&T, ^177^Lu-PSMA-D4, ^177^Lu-PSMA-R2, ^64/67^Cu-RPS-085, and ^161^Tb-PSMA-617 have been developed, with ^177^Lu-PSMA-617 and ^177^Lu-PSMA I&T showing the most promise [140,141,142,143]. ^177^Lu-PSMA-617, with its high affinity for PSMA, specifically targets prostate cancer cells and exhibits superior biodistribution and safety compared to ^177^Lu-PSMA-I&T. Clinical studies have demonstrated remarkable efficacy of this approach in prostate cancer management, with predictive models incorporating age and hemoglobin parameters enabling treatment response stratification [141,144,145]. While both ^177^Lu-PSMA-617 and ^177^Lu-PSMA-I&T have established safety profiles and therapeutic effectiveness in prostate cancer, optimization of their pharmacokinetic characteristics remains an important area for further development [146,147]. To further enhance tumor uptake and retention time of radiotherapeutic agents, structural modifications have introduced albumin-binding groups, resulting in long-circulating agents such as ^177^Lu-Alb-L4 and ^177^Lu-Alb-L6 [148]. These albumin-binding groups can be combined with affinity-modifying groups to generate improved radiotherapeutic agents, such as ^177^Lu-HTK03121 and ^177^Lu-HTK03123 [149]. Kuo et al. have also replaced glutamic acid in PSMA-targeting pharmacophores with analogous structures to develop ^177^Lu-HTK03149 [150]. Additionally, a pre-targeting strategy based on inverse electron demand Diels-Alder (iEDDA) reactions has been used to synthesize clickable chelators and radiolabeled drugs, such as ^90^Y-DOTA-Tz [151]. These approaches significantly improve tumor uptake and enhance therapeutic efficacy. Huangfu et al. developed a dual-receptor targeting peptide (Sigma-1 receptor/PSMA-P) that targets both the Sigma-1 receptor (S1R) and PSMA, labeled with ^68^Ga or ^177^Lu. This approach significantly enhances the targeting and therapeutic specificity in prostate cancer [152]. To address the issue of hematologic toxicity associated with full antibodies in radioimmunotherapy, a smaller mini-antibody targeting prostate stem cell antigen (PSCA), ^131^I-A11Mb, has been developed. Its smaller size allows for faster kinetics, minimal off-target toxicity, and good therapeutic potential in targeted radioimmunotherapy [4]. Bratanovic et al. targeting the gastrin-releasing peptide receptor (GRPR), overexpressed in some solid malignancies, synthesized a new bombesin-like derivative, ProBOMB2, and developed ^68^Ga/^177^Lu-ProBOMB2 for diagnostic and therapeutic applications [153]. Additionally, for neuroendocrine prostate cancer (NEPC), which currently has limited treatment options and poor prognosis, a radioactive antibody targeting DLL3, ^177^Lu-DTPA-SC16, has been successfully developed, showing high tumor uptake and significant therapeutic efficacy [5].

Compared to targeted β-therapy, targeted α-therapy (TAT) delivers potent local radiation to cancer cells and the tumor microenvironment more selectively, minimizing toxicity while controlling cancer [154,155,156]. Radium-223 dichloride (radium-223, Xofigo^®^) is the first α-particle emitter approved for treating bone metastases in mCRPC [155]. However, the therapeutic efficacy of ^223^Ra-dichloride is restricted to osseous metastases due to its calcium-mimetic properties and preferential incorporation into bone remodeling sites [157,158]. In response, Juzeniene et al. have explored combining ^223^Ra with other cytotoxic agents, androgen receptor-targeted drugs, or immuno-oncology therapies, while also focusing on developing new agents by conjugating other α-emitting isotopes with PSMA-targeting ligands [159]. As a short half-life TAT radionuclide, ^213^Bi has shown in preclinical studies that its PSMA-I&T or JVZ-008 nanobody conjugates effectively target tumors and inhibit xenograft tumor growth. However, its dosimetric disadvantages limit its clinical application [160,161]. PSMA-targeting molecule PSMA 6, labeled with ^211^At, significantly extended survival in animal models. However, the high uptake of ^211^At-PSMA 6 in the proximal tubules of the kidney led to late-stage nephrotoxicity (≤12 months) [162]. PSMA-617 and PSMA-I&T labeled with ^225^Ac have shown significant efficacy in mCRPC patients, but salivary gland toxicity (xerostomia) has become a dose-limiting factor [163,164,165,166,167]. Improvement strategies include using the antibody J591 instead of small-molecule ligands or optimizing dosing regimens to reduce salivary gland/kidney distribution [168,169,170]. Other radionuclides, such as ^227^Th, form highly stable ^227^Th-targeted conjugates (TTCs) when chelated (3,2-HOPO) with antibodies or other targeting molecules [171], while ^149^Tb offers both α-therapy and PET imaging potential [172,173]. Dual α-therapy (^224^Ra & ^212^Pb), combining bone targeting and PSMA targeting, can simultaneously address bone metastasis and soft tissue lesions [174]. Furthermore, to address the issue of inadequate response to ^225^Ac-PSMA-617 in 20% of patients, combination therapies (such as low-activity ^225^Ac with ^177^Lu, PARP inhibitors, or PD-1 blockers) can enhance efficacy and reduce toxicity through synergistic mechanisms, with preclinical models confirming their potential [165,170,175,176,177].

Radionuclides emitting auger electrons (AEs) feature low energy (0.02–50 keV), short range (0.0007–40 µm), and high linear energy transfer (1–10 keV/µm), making them potentially pivotal in targeted radionuclide therapy for metastatic and diffuse diseases. ^165^Er is a pure AE-emitting radionuclide with chemical properties similar to the clinically used radionuclide ^177^Lu. Silva et al. have developed new biomedical cyclotron irradiation and radiochemical separation methods for efficiently producing ^165^Er. They used this isotope to synthesize a novel PSMA-targeting radiopharmaceutical, ^165^Er-PSMA-617, providing a new tool for basic research in targeted radionuclide therapy [178].

### 7.2. Breast Cancer

Breast cancer (BC) represents one of the most prevalent malignancies among female populations, and its incidence has been increasing annually in China. Approximately 20% to 25% of invasive breast cancers overexpress human epidermal growth factor receptor 2 (HER2), making it an effective target for radioimmunotherapy (RIT) [179]. Trastuzumab, an FDA-approved monoclonal antibody (mAb), targets the extracellular domain of HER2 and induces antibody-dependent cellular cytotoxicity to kill tumor cells [180]. Based on this, ^177^Lu-CHX-A″-DTPA-Trastuzumab has been developed, which induces dose-dependent cytotoxicity through apoptosis, providing a potential treatment for HER2-positive BC [6]. However, due to the large size, slow pharmacokinetics, and poor tumor penetration of trastuzumab, one research group [3] attempted to use pepsin to cleave the full antibody into bivalent F(ab′)_2_ fragments. This led to the development of a potential therapeutic agent, ^177^Lu-CHX-A″-DTPA-F(ab′)_2_-Trastuzumab, which exhibits similar cytotoxicity in terms of membrane damage, apoptosis induction, and cell death compared to ^177^Lu-CHX-A″-DTPA-Trastuzumab. However, its in vivo efficacy requires further investigation. In addition to enzyme-digesting full antibodies, radiolabeled peptides such as DOTA-A9 and its PEGylated variant (^177^Lu-DOTA-PEG_4_-A9), as well as leuprolide peptide (^68^Ga/^177^Lu-leuprolide), have been developed for potential use in early detection and targeted therapy of breast cancer [89,181]. Additionally, trastuzumab has limitations in treating metastatic breast cancer. Firstly, these are related to the ligand-dependent dimerization of the HER2 receptor in the extracellular domain-II (ECD-II). Combining trastuzumab with pertuzumab (^177^Lu-DOTA-pertuzumab) effectively addresses this issue [7]. Secondly, trastuzumab is not effective for treating HER2-positive brain metastases. Therefore, single-domain antibody fragments (sdAb) 2Rs15d, labeled with ^111^In, ^225^Ac, or ^131^I, have been developed, showing potential as a valuable adjunct therapy for refractory HER2-positive metastatic cancer patients [2]. To address the issue of lymph node metastasis in breast cancer, Zhu et al. [182] developed a multifunctional nanoprobe (NP-mAb) based on upconversion nanoparticles (NaGdF_4_:Yb, Tm@NaLuF_4_), which is modified with PEG and anti-HER2 monoclonal antibody (trastuzumab) for efficient targeting and enrichment of lymph node metastases. The bisphosphonate groups of NP-mAb can chelate both ^68^Ga and ^177^Lu, enabling integrated diagnostic and therapeutic applications.

Triple-negative breast cancer (TNBC) is the most aggressive subtype of breast cancer, and there remains an unmet need for effective treatment. ^177^Lu-NM600, developed based on tumor-targeting alkylphosphocholine (NM600), exhibits excellent tumor targeting, good tolerance, and significant potential to inhibit tumor growth and extend survival in preclinical models. It has been successfully used in targeted radionuclide therapy for TNBC [13]. Additionally, nanoparticles co-loaded with the chemotherapy drug (doxorubicin) and ^177^Lu (FA-PDA@mSiO_2_) enable spatiotemporal synergistic radio-chemotherapy, increasing the complete remission rate of tumors to 65% [17]. A separate research group successfully conjugated the anti-epidermal growth factor receptor (EGFR) antibody nimotuzumab via SpyTag technology for α-radioimmunotherapy applications. In preclinical studies, the ^225^Ac-labeled nimotuzumab-SpyTag-∆N-SpyCatcher construct demonstrated significant survival benefit in murine models of EGFR-positive triple-negative breast cancer [8]. Collectively, these innovative strategies offer promising therapeutic avenues for aggressive, treatment-resistant breast cancer subtypes.

Primary neuroendocrine breast cancer is relatively rare compared to TNBC. For this type of breast cancer, Liu et al. have developed a radiolabeled somatostatin analogue, ^68^Ga/^177^Lu-DOTATOC, which has shown significant therapeutic efficacy [183].

### 7.3. Neuroendocrine Tumors

Neuroendocrine tumors (NETs) are relatively rare tumors, primarily occurring in the gastrointestinal tract, pancreas, and lungs, with overexpression of somatostatin receptors (SSTR) [9]. Targeted radiopharmaceuticals utilizing somatostatin analogs (SSAs) for peptide receptor radionuclide therapy comprise several clinically established agents, including ^111^In-pentetreotide [184], ^90^Y-DOTATOC [185], and ^177^Lu-DOTATATE [28]. Among them, ^177^Lu-DOTATATE is the first FDA-approved peptide receptor radionuclide therapy for GEP-NENs, significantly extending progression-free survival (PFS) by targeting SSTR [186,187]. Building on this, a radiolabeled SSA modified with Evans blue (^177^Lu-DOTA-EB-TATE) has been developed, demonstrating better therapeutic efficacy in treating advanced metastatic neuroendocrine tumors compared to ^177^Lu-DOTATATE [188]. The use of radiolabeled SSAs for therapy shows broad potential, not only for well-differentiated GEP-NENs but also for inoperable low-grade bronchial neuroendocrine tumors, inoperable or progressive pheochromocytomas, paragangliomas, and medullary thyroid cancer [189].

In recent years, the use of radiolabeled somatostatin receptor antagonists has gradually emerged as a novel therapy for neuroendocrine tumors. Antagonists have lower receptor desensitization and internalization, allowing them to bind to more sites, resulting in higher tumor uptake than their corresponding agonists [9]. For this therapy, promising therapeutic combinations include ^68^Ga-DOTA-JR11 and ^177^Lu-DOTA-JR11, ^68^Ga-NODAGA-JR11 and ^177^Lu-DOTA-JR11, and ^68^Ga-DOTA/NODAGA-LM3 and ^177^Lu-DOTA-LM3 [125,126,190].

In addition to SSTR, norepinephrine transporter (NET) is also overexpressed in neuroendocrine tumors. The radiopharmaceutical ^131^I-labeled meta-iodobenzylguanidine (^131^I-mIBG) has received FDA approval for therapeutic use in locally advanced or metastatic pheochromocytoma and paraganglioma cases where systemic anticancer therapy is indicated [191,192].

### 7.4. Glioma

Glioma originates from glial cells and is the most common primary intracranial tumor [193]. EGFR is known to be overexpressed in various solid tumors, including gliomas. Based on this, Sobral et al. synthesized radiolabeled EEEEYFELV peptide and its analog (DEDEYFELV) targeting EGFR. It was found that both ^131^I-labeled peptides exhibited high specificity for EGFR, favorable biodistribution, and promising potential for use in glioma cells [194].

Glioblastoma multiforme (GBM) is the most malignant and prognostically poor type of glioma, characterized by high proliferative and invasive abilities. It is also resistant to conventional radiotherapy and chemotherapy, highlighting the urgent need for novel treatment options. In addition to EGFR, GBM also overexpresses α_v_β_3_ integrin. Radiolabeled arginine-glycine-aspartate (RGD) analogs (GRGDYV and GRGDHV) specifically bind to integrin α_v_β_3_, demonstrating good radiolabeling efficiency, stability, and binding and internalization capabilities in GBM cells, with potential clinical application value [195]. In addition to single-receptor-targeting peptides, a bispecific heterodimeric peptide targeting both vascular endothelial growth factor receptor (VEGFR) and integrin α_V_β_3_ (iRGD-C6-lys(^211^At-ATE)-C6-^D^A7R) has been developed. This peptide significantly inhibits tumor growth and extends survival without noticeable liver or kidney toxicity [10]. Substance P (SP) is a natural ligand for the NK-1 receptor, which is highly expressed on the cell membrane of GBM and in tumor neovasculature. Intratumoral injection of ^213^Bi-DOTA-SP or ^225^Ac-DOTAGA-SP has been introduced as a TAT for the treatment of recurrent GBM [196]. Silva et al. [197] developed a novel type of gold nanoparticle (AuNP-SP and AuNP-SPTyr8), which carries DOTA chelators and SP, enhancing tumor cell endocytosis and demonstrating its potential as a therapeutic strategy for GBM.

### 7.5. Melanoma

In the systemic treatment of metastatic melanoma, targeted radionuclide therapy represents a significant clinical advancement in the field of adjuvant therapy. Preclinical studies and multiple clinical trials have confirmed the effectiveness of peptide- or antibody-targeted radiotherapy in treating melanoma [198]. Peptides targeting melanocortin 1 receptor (MC1-R) using α- and β-emitting radiopharmaceuticals have been labeled with radiolabels and shown effective melanoma growth control in mouse melanoma models [199]. However, dose-limiting toxicity has hindered the clinical translation of these therapies at the doses required for tumor eradication [200]. To optimize therapeutic outcomes in melanoma through targeted radionuclide therapy, a research team [15] developed ^177^Lu-labeled ultrasmall fluorescent core-shell silica nanoparticles and designed PEGylated Cornell prime dots (C′ dots) to target overexpressed MC1-R. The novel drug (^177^Lu-DOTA-αMSH-PEG-Cy5-C′dots) demonstrated favorable biodistribution and therapeutic efficacy, significantly improving melanoma treatment and extending survival, while overcoming the biological properties and dose-limiting toxicity of existing single-molecule therapies. Additionally, the ^89^Zr/^225^Ac radionuclides for radiolabeled submicrometric core-shell particles (SPs) have been developed, aiming to integrate diagnosis and therapy for melanoma. In the ^89^Zr/^225^Ac-SPs, the long half-life of ^89^Zr supports multi-time-point biodistribution studies, while the α-particles of ^225^Ac effectively kill local tumors, enhancing the radiopharmaceutical treatment efficiency for melanoma [132].

The radionuclide-targeted drugs used for the aforementioned tumor types are summarized (Table 2).

## 8. Challenges and Future Directions

Radiolabeled biomaterials show significant potential in cancer therapy, but they still face multiple challenges in terms of technical optimization, clinical translation, and the development of innovative strategies. This section will systematically review current bottlenecks and clinical translation challenges, and propose future development recommendations based on the latest research advancements.

### 8.1. Technical Challenges

The large-scale application of high-activity isotopes (such as α-emitting ^225^Ac and β-emitting ^177^Lu) in radiolabeled biomaterials is severely limited by their scarcity and the complexity of production. For instance, although the high linear energy transfer (LET) characteristics of ^225^Ac make it a promising isotope for precision therapy, its production relies on limited reactors, and the vulnerability of its supply chain makes it difficult to meet clinical demands [201]. Moreover, the targeting and stability of radiolabeled biomaterials are crucial factors affecting therapeutic outcomes. The retention of sub-isotopes from ^225^Ac may cause radiation damage to non-target organs, further limiting its long-term application [202]. On the other hand, degradation products of nanocarriers may induce inflammatory responses [203], and the charge properties of the nanomaterials themselves may interfere with cellular death mechanisms [204]. Studies have shown that the modification of nanocarriers can effectively reduce their toxicity to normal cells while improving their efficiency in targeting tumor cells. The design and application of hybrid nanocarriers hold promise in addressing common issues faced by traditional nanocarriers, providing a new breakthrough for the development of radiolabeled therapies [205].

### 8.2. Challenges in Clinical Translation

Clinical translation of radiolabeled biomaterials faces challenges related to biosafety and long-term toxicity. The radiotoxicity of isotopes may lead to severe side effects such as hemolytic anemia and myelodysplastic syndromes (MDSs), limiting their long-term application [206]. Developing effective biological barriers (e.g., isotope encapsulation technology) and toxicity mitigation strategies (e.g., stem cell transplantation) is crucial to addressing this issue. For example, ^225^Ac-labeled core-shell particles reduce non-target organ damage through biocompatible coatings, though the long-term migration risk of sub-isotopes still requires further validation [132]. Additionally, long-term toxicity and the lack of biodistribution data limit the clinical translation of radiolabeled materials.

Radiolabeled therapy involves multiple disciplines, including nuclear medicine, materials science, oncology, and radiobiology, with insufficient interdisciplinary collaboration being a major factor limiting the efficiency of technology translation. For example, in PRRT, while ^177^Lu-DOTATATE has been FDA-approved for neuroendocrine tumors, its Phase III clinical trial data remains insufficient, making it difficult to determine the optimal dosing regimen and long-term complication risks [207]. Establishing multidisciplinary collaboration platforms and standardized research processes (e.g., unified dosage evaluation and biodistribution analysis methods) through artificial intelligence technologies is key to improving translation efficiency.

### 8.3. Future Directions

To overcome current challenges and advance the application of radiolabeled biomaterials in cancer therapy, future research should focus on the following directions:

#### 8.3.1. Development of Novel Isotopes and Labeling Techniques

The development of highly active, low-toxicity isotopes is a key direction to enhance therapeutic efficacy and reduce side effects. Additionally, improving isotope labeling techniques can enhance labeling efficiency and stability [208]. For example, Falco et al. reported a click chemistry-based 225Ac labeling method that significantly improved labeling efficiency and reduced off-target effects [209]. Furthermore, the development of multifunctional carriers for radiolabeling can further enhance tumor targeting and therapeutic efficacy [137].

#### 8.3.2. Multifunctional Combined Treatment Strategies and Theranostics-Immuno/Gene Synergy

Multifunctional combined strategies, such as radiotherapy–immunotherapy, radiotherapy–chemotherapy, and radiotherapy–photothermal therapy, are important research directions for the future. For example, one research group successfully achieved a synergistic effect between radiolabeled radioactive therapy and immunotherapy by combining ^131^I-labeled catalase with sodium alginate [210]. Additionally, Jing et al. combined the commonly used photosensitizer indocyanine green with ^177^Lu-labeled metal-ceramics to construct a multifunctional nanoplatform, offering new insights into combining radiotherapy and photothermal therapy [211]. These studies demonstrate the tremendous potential of multifunctional combination strategies to improve therapeutic efficacy and reduce side effects, laying the foundation for future innovations in cancer treatment. Furthermore, immunotherapy and gene therapy can be strategically integrated with theranostics to establish multidimensional synergistic mechanisms. Utilizing radionuclide tracing technology enables both precise lesion targeting and real-time therapeutic monitoring, while concurrent immunomicroenvironment-specific activation and precise gene regulatory network modulation achieve spatiotemporal-precise therapeutic intervention.

#### 8.3.3. Application of Artificial Intelligence and Big Data

The integration of artificial intelligence (AI) and big data analytics into radiopharmaceutical therapy shows significant potential to optimize treatment efficacy while minimizing adverse effects. AI can be used to optimize isotope dosages, predict tumor targeting, and assess potential toxicity. Currently, research groups have developed deep learning models to optimize the dosing regimen of ^177^Lu-DOTATATE and reduce bone marrow toxicity [212]. In the future, real-time imaging data (e.g., PET/CT) can be integrated to develop a dynamic dose regulation system for precise intervention throughout the entire treatment process [213]. The integration of spatial transcriptomics with computational modeling holds promise for optimizing personalized theranostic strategies using radionuclide-labeled biomaterials [214].

## 9. Conclusions

Radionuclide-labeled biomaterials demonstrate immense potential in the field of tumor-targeted therapy. Their advantages, including precise delivery, localized high-dose radiation, and the ability to overcome drug resistance, offer patients new treatment options. However, challenges such as radionuclide availability and stability, limitations of carrier systems, and barriers to clinical translation remain significant hurdles. Moving forward, the development of novel radionuclides and efficient labeling techniques, the optimization of multifunctional targeted delivery systems, the promotion of multimodal combined treatment strategies, and the integration of artificial intelligence will be critical in overcoming these technical barriers. Additionally, fostering multidisciplinary collaboration among nuclear medicine, materials science, and oncology will accelerate the clinical application and dissemination of radionuclide-labeled biomaterials. Through continuous innovation and resource integration, this technology holds promise for providing more effective and safer solutions for precise tumor treatment, bringing renewed hope to patients.

## Figures and Tables

**Table 1:** Basic properties of individual radionuclides.

Radionuclide	Type	t_1/2_	Production	Labeling	Application	Ref.
^225^Ac	α	9.9 d	Accelerator, Reactor	chelating agent	Prostate cancer, neuroendocrine tumors, melanoma	[78,79,80,81,88]
^211^At	α	7.21 h	Accelerator	substitution	Gliomas, prostate cancer	[49,53,54,55]
^213^Bi	α	45.6 m	^225^Ac decay chain	chelating agent	Neuroendocrine tumors, glioblastoma	[55,58,61]
^223^Ra	α	11.4 d	^227^Ac decay chain, uranium extraction	macrocyclic ligand	Bone metastatic prostate cancer	[45,46,47,48]
^177^Lu	β^−^	6.64 d	Reactor	chelating agent	Neuroendocrine tumors, prostate cancer, breast cancer	[23,24,25,26,27]
^90^Y	β^−^	64 h	Reactor	chelating agent	Non-Hodgkin’s lymphoma, liver cancer (microsphere therapy)	[29,30,31,32,33,34,35,36]
^131^I	β^−^	8.02 d	Neutron irradiation ^130^Te, uranium fission	substitution	Thyroid cancer, neuroblastoma	[37,38,41,42]
^161^Tb	β^−^, AE	6.9 d	Accelerator, Reactor	HEDP, liposomes, antibody coupling	Tumor therapy targeting DNA	[73,75,76,77]
^125^I	AE	59.4 d	Neutron capture by Xe	substitution	Prostate cancer, eye cancer	[41,65,66,67,68,69,70,71,72]

**Table 2:** Application of radiopharmaceuticals in different tumor types for targeted radionuclide therapy.

Classification of Tumors	Therapeutic Radiation Type	Radiopharmaceuticals	Ref.
Prostate Cancer	Beta	^177^Lu-PSMA-617, ^177^Lu-PSMA I&T, ^177^Lu-PSMA-D4, ^177^Lu-PSMA-R2, ^64/67^Cu-RPS-085, ^161^Tb-PSMA-617, ^177^Lu-Alb-L4, ^177^Lu-Alb-L6, ^177^Lu-HTK03121, ^177^Lu-HTK03123, ^177^Lu-HTK03149, ^90^Y-DOTA-Tz, ^68^Ga/^177^Lu-S1R/PSMA-P, ^131^I-A11Mb, ^68^Ga/^177^Lu-ProBOMB2, ^177^Lu-DTPA-SC16	[4,5,140,141,142,143,144,145,146,147,148,149,150,151,152,153]
	Alpha	^223^Ra (Xofigo^®^), ^213^Bi-PSMA-I&T, ^213^Bi-PSMA-JVZ-008, ^211^At-PSMA 6, ^225^Ac-PSMA-617, ^225^Ac-PSMA-I&T, ^225^Ac-J591, ^227^Th-PSMA-TTC, ^149^Tb-PSMA-617, ^212^Pb-NG001	[155,160,161,162,163,164,165,166,167,168,169,170,171,172,173,174,175,176,177].
	Auger Electrons	^165^Er-PSMA-617	[178]
Breast Cancer	Beta	^177^Lu-CHX-A″-DTPA-Trastuzumab, ^177^Lu-CHX-A″-DTPA-F(ab’)_2_-Trastuzumab, ^177^Lu-DOTA-PEG_4_-A9, ^68^Ga/^177^Lu-Leuprolide, ^177^Lu-DOTA-Pertuzumab, ^68^Ga/^177^Lu-NP-Trastuzumab (NaGdF_4_:Yb, Tm@NaLuF_4_), ^177^Lu-NM600, ^177^Lu-FA-PDA@mSiO_2_ (Co-loaded with Doxorubicin), ^68^Ga/^177^Lu-DOTATOC	[3,6,7,13,17,89,181,182,183]
	Alpha	^225^Ac-Nimotuzumab-SpyTag-∆N-SpyCatcher	[8]
	Alpha and Beta	^111^In/^225^Ac/^131^I-2Rs15d (sdAb)	[2]
Neuroendocrine Tumor	Beta	^90^Y-DOTATOC, ^177^Lu-DOTATATE, ^177^Lu-DOTA-EB-TATE, ^68^Ga/^177^Lu-DOTA-JR11, ^68^Ga-NODAGA/^177^Lu-DOTA-JR11, ^68^Ga/^177^Lu-DOTA/NODAGA-LM3, ^131^I-mIBG	[28,125,126,185,186,187,188,190,191,192]
	Auger Electrons	^111^In-Pentetreotide	[184]
Glioma	Beta	^131^I-EEEEYFELV, ^131^I-DEDEYFELV, ^131^I-GRGDYV/^99m^Tc-GRGDHV, ^67^Ga/^177^Lu-AuNP-SP/SPTyr8	[194,195,197]
	Alpha	iRGD-C6-lys(^211^At-ATE)-C6-^D^A7R, ^213^Bi-DOTA-SP, ^225^Ac-DOTAGA-SP	[10,196]
Melanoma	Beta	^177^Lu-DOTA-αMSH-PEG-Cy5-C′ Dots	[15]
	Alpha	^89^Zr/^225^Ac-SPs	[132]
	Alpha, Beta, Auger Electrons	^111^In, ^67/68^Ga, ^64^Cu, ^90^Y, ^212^Pb, ^99m^Tc, ^188^Re-αMSH Peptides	[199]

## Data Availability

No new data were created or analyzed in this study. Data sharing is not applicable to this article.

## Abbreviations

The following abbreviations are used in this manuscript:

TRT	Targeted Radionuclide Therapy
LET	Linear Energy Transfer
PRRT	Peptide Receptor Radionuclide Therapy
SSTR	Somatostatin Receptor
PSMA	Prostate-Specific Membrane Antigen
HER2	Human Epidermal Growth Factor Receptor 2
EGFR	Epidermal Growth Factor Receptor
VEGFR	Vascular Endothelial Growth Factor Receptor
NET	Norepinephrine Transporter
mCRPC	Metastatic Castration-Resistant Prostate Cancer
TNBC	Triple-Negative Breast Cancer
GEP-NENs	Gastroenteropancreatic Neuroendocrine Neoplasms
GBM	Glioblastoma Multiforme
MDS	Myelodysplastic Syndromes
PFS	Progression-Free Survival
ICD	Immunogenic Cell Death
DOTA	1,4,7,10-Tetraazacyclododecane-1,4,7,10-tetraacetic acid
DTPA	Diethylenetriaminepentaacetic acid
NOTA	1,4,7-Triazacyclononane-1,4,7-triacetic acid
HEHA	Hexaazacyclohexadecane-N,N′,N′′,N′′′,N′′′′,N′′′′′-hexaacetic acid
EDTA	Ethylenediaminetetraacetic acid
CHX-DT	Cyclohexyl-diethylenetriaminepentaacetic acid
PA	
PEPA	p-isothiocyanatobenzyl-3,6,9,15-tetraazabicyclo[9.3.1]pentadeca-1(15),11,13-triene-3,6,9-triacetic acid
DOTP	1,4,7,10-Tetraazacyclododecane-1,4,7,10-tetramethylenephosphonic acid
LPy	Lipoyl-Pyridine
Macropa	Macrocyclic Pyridinophane
SPECT	Single-Photon Emission Computed Tomography
PET	Positron Emission Tomography
MRI	Magnetic Resonance Imaging
PAI	Photoacoustic Imaging
PTT	Photothermal Therapy
PDT	Photodynamic Therapy
RBE	Relative Biological Effectiveness
RCY	Radiochemical Yield
BFC	Bifunctional Chelator
scFv	Single-Chain Variable Fragment
mAb	Monoclonal Antibody
F(ab′)_2_	Fragment antigen-binding
sdAb	Single-Domain Antibody
EV	Extracellular Vesicle
AuNPs	Gold Nanoparticles
PIONs	Porous Iron Oxide Nanoparticles
MPDA	Mesoporous Polydopamine
SWNT	Single-Walled Carbon Nanotube
PEG	Polyethylene Glycol
iRGD	Internalizing RGD
NK-1	Neurokinin-1
MC1-R	Melanocortin 1 Receptor
S1R	Sigma-1 Receptor
GRPR	Gastrin-Releasing Peptide Receptor
PSCA	Prostate Stem Cell Antigen
DLL3	Delta-Like Ligand 3
FRα	Folate Receptor Alpha
CD133	Cluster of Differentiation 133
α_v_β_3_	Integrin Alpha-V Beta-3
DAMPs	Damage-Associated Molecular Patterns
FDA	Food and Drug Administration
AI	Artificial Intelligence
SIRT	Selective Internal Radiation Therapy
PMMA	Polymethylmethacrylate
EBRT	External Beam Radiation Therapy
SELEX	Systematic Evolution of Ligands by Exponential Enrichment
